# Characterization of a second secologanin synthase isoform producing both secologanin and secoxyloganin allows enhanced *de novo* assembly of a *Catharanthus roseus* transcriptome

**DOI:** 10.1186/s12864-015-1678-y

**Published:** 2015-08-19

**Authors:** Thomas Dugé de Bernonville, Emilien Foureau, Claire Parage, Arnaud Lanoue, Marc Clastre, Monica Arias Londono, Audrey Oudin, Benjamin Houillé, Nicolas Papon, Sébastien Besseau, Gaëlle Glévarec, Lucia Atehortùa, Nathalie Giglioli-Guivarc’h, Benoit St-Pierre, Vincenzo De Luca, Sarah E. O’Connor, Vincent Courdavault

**Affiliations:** Université François-Rabelais de Tours, EA2106 “Biomolécules et Biotechnologies Végétales”, UFR Sciences et Techniques, 37200 Tours, France; Universidad de Antioquia, Laboratorio de Biotecnología, Sede de Investigación Universitaria, Medellín, Colombia; Department of Biological Sciences, Brock University, 500 Glenridge Avenue, St Catharines, Ontario L2S 3A1 Canada; Department of Biological Chemistry, John Innes Centre, Norwich Research Park, Colney, Norwich, NR4 7UH UK

**Keywords:** *Catharanthus roseus*, Transcriptome assembly, Isoform, Secologanin synthase, Secoxyloganin

## Abstract

**Background:**

Transcriptome sequencing offers a great resource for the study of non-model plants such as *Catharanthus roseus*, which produces valuable monoterpenoid indole alkaloids (MIAs) via a complex biosynthetic pathway whose characterization is still undergoing. Transcriptome databases dedicated to this plant were recently developed by several consortia to uncover new biosynthetic genes. However, the identification of missing steps in MIA biosynthesis based on these large datasets may be limited by the erroneous assembly of close transcripts and isoforms, even with the multiple available transcriptomes.

**Results:**

Secologanin synthases (SLS) are P450 enzymes that catalyze an unusual ring-opening reaction of loganin in the biosynthesis of the MIA precursor secologanin. We report here the identification and characterization in *C. roseus* of a new isoform of SLS, SLS2, sharing 97 % nucleotide sequence identity with the previously characterized SLS1. We also discovered that both isoforms further oxidize secologanin into secoxyloganin. SLS2 had however a different expression profile, being the major isoform in aerial organs that constitute the main site of MIA accumulation. Unfortunately, we were unable to find a current *C. roseus* transcriptome database containing simultaneously well reconstructed sequences of SLS isoforms and accurate expression levels. After a pair of close mRNA encoding tabersonine 16-hydroxylase (T16H1 and T16H2), this is the second example of improperly assembled transcripts from the MIA pathway in the public transcriptome databases. To construct a more complete transcriptome resource for *C. roseus*, we re-processed previously published transcriptome data by combining new single assemblies. Care was particularly taken during clustering and filtering steps to remove redundant contigs but not transcripts encoding potential isoforms by monitoring quality reconstruction of MIA genes and specific SLS and T16H isoforms. The new consensus transcriptome allowed a precise estimation of abundance of SLS and T16H isoforms, similar to qPCR measurements.

**Conclusions:**

The *C. roseus* consensus transcriptome can now be used for characterization of new genes of the MIA pathway. Furthermore, additional isoforms of genes encoding distinct MIA biosynthetic enzymes isoforms could be predicted suggesting the existence of a higher level of complexity in the synthesis of MIA, raising the question of the evolutionary events behind what seems like redundancy.

**Electronic supplementary material:**

The online version of this article (doi:10.1186/s12864-015-1678-y) contains supplementary material, which is available to authorized users.

## Background

Monoterpenoid Indole Alkaloids (MIAs) constitute a remarkable class of specialized metabolites, with a huge chemical diversity, and a source of several active compounds, including important pharmacophores. Some of the most active anticancer drugs are based on this type of skeleton including camptothecans and Vinca alkaloids. The later compounds are present in minute amounts in the leaves of the Madagascar periwinkle, *Catharanthus roseus*, and result from a complex metabolic pathway, which is the target of expanding research efforts in the phytochemical genomic era [1, 2].

**Fig. 1:**
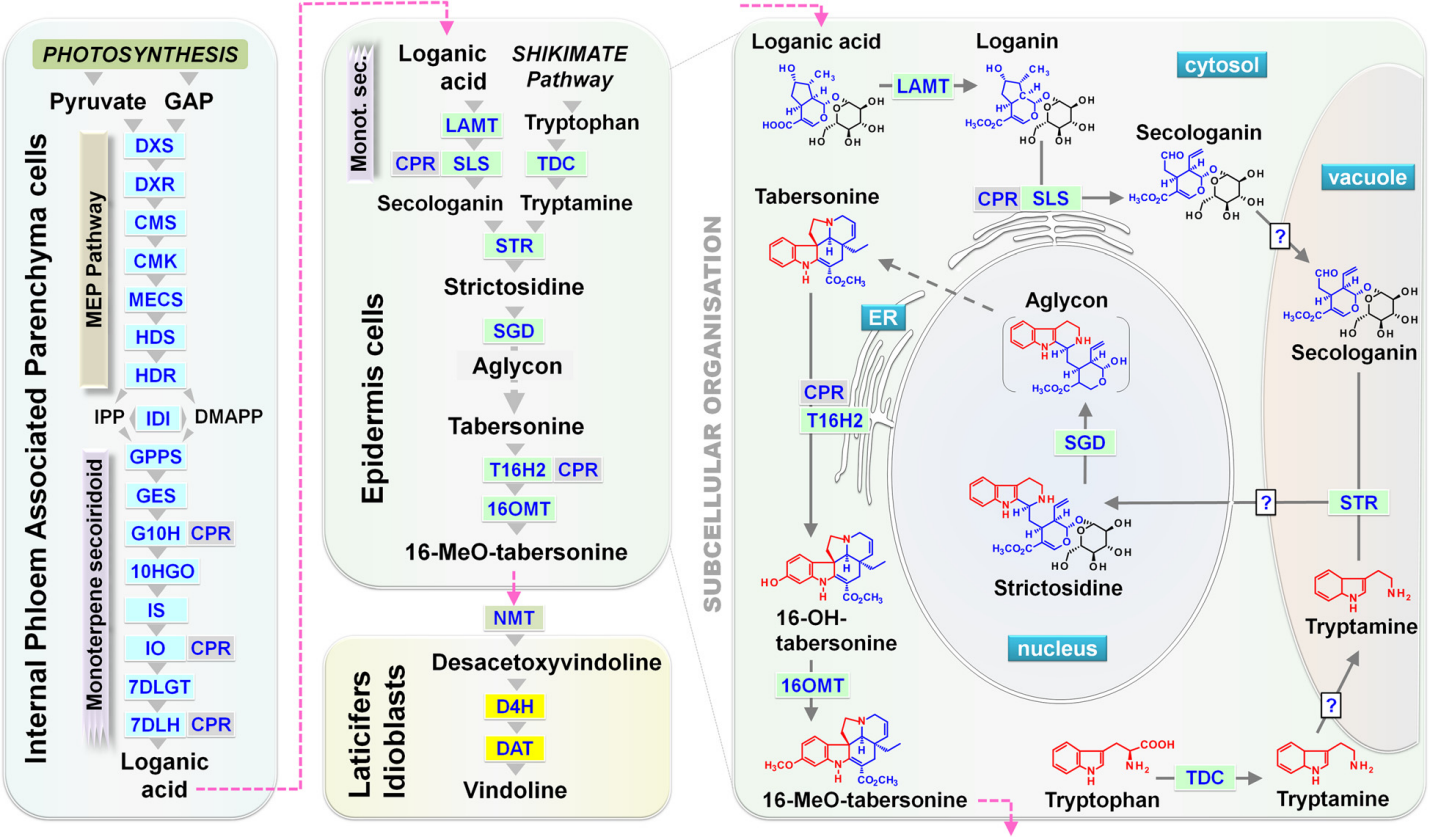
The biosynthetic pathway of MIA in *C. roseus* leaves. Simplified representation of the MIA biosynthesis in *C. roseus* highlighting the subcellular organization of the central steps of the pathway. Known single enzymatic steps in each cell type are indicated by grey arrows and abbreviation of enzyme names. Broken grey arrows and broken pink arrows indicate unknown enzymatic steps and metabolite translocation, respectively. DXS, 1-deoxy-D-xylulose-5-phosphate (DXP) synthase; DXR, DXP reductoisomerase; CMS, 4-(cytidine 5′-diphospho)-2*C*-methyl-D-erythritol (CM) synthase; CMK, CM kinase; MECS, 2*C*-methyl-D-erythritol-2,4-cyclodiphosphate (MEC) synthase; HDS, hydroxymethylbutenyl 4-diphosphate (HD) synthase; HDR, HD reductase; IDI, isopentenyl diphosphate isomerase; GPPS, geranyl diphosphate synthase; GES, geraniol synthase; G10H (CYP76B6), geraniol 10-hydroxylase; CPR, cytochrome P450-reductase; 10HGO, 10-hydroxygeraniol oxidoreductase; IO, iridoid oxidase; IS, iridoid synthase; 7DLGT, 7-deoxyloganetic acid glucosyltransferase; 7DLH, 7-deoxyloganic acid 7-hydroxylase; LAMT, loganic acid *O*-methyltransferase; SLS (CYP72A1), secologanin synthase; TDC, tryptophan decarboxylase; STR, strictosidine synthase; SGD, strictosidine β-glucosidase; T16H2 (CYP71D351), tabersonine 16-hydroxylase 2; 16OMT, 16-hydroxytabersonine *O*-methyltransferase; NMT, 16-methoxy-2,3-dihydrotabersonine N-methyltransferase; D4H, desacetoxyvindoline 4-hydroxylase; DAT, deacetylvindoline 4-*O*-acetyltransferase. DMAPP, dimethylallyl diphosphate; GAP, glyceraldehyde 3-phosphate; IPP, isopentenyl diphosphate

MIAs stem from a unique polyvalent skeleton named strictosidine. This central precursor is the condensation product of a tryptophan-derived amine coupled to an extensively modified monoterpenoid moiety (Fig. 1). While tryptamine is derived from tryptophan by a single reaction catalyzed by tryptophan decarboxylase (TDC) [3], the assembly of the monoterpene secoiridoid moiety, requires several reactions to convert the methyl-erythritol phosphate (MEP) pathway-derived monoterpenoid skeleton into secologanin (Fig. 1) [4].

Recently, the elusive reaction scheme of secologanin biosynthesis has been elucidated in *C. roseus*. In the plastid-localized MEP pathway, glyceraldehyde 3-phosphate (GAP) and pyruvate are converted into the universal isoprenoid precursors, isopentenyl diphosphate (IPP) and dimethylallyl diphosphate (DMAPP), through seven enzymatic reactions. The subsequent conversion of these primary metabolites into secologanin, by the monoterpene secoiridoid pathway, requires ten more enzymes.

First, the prenyl-transfer of IPP on DMAPP by geranyl diphosphate synthase (GPPS) [5], is followed by formation of the monoterpene geraniol by geraniol synthase (GES) [6] (Fig. 1). Subsequently, geraniol is hydroxylated into the diol 10-hydroxygeraniol (alternative nomenclature: 8-hydroxygeraniol) and further oxidized into 10-oxogeraniol by the bifunctional P450 CYP76B6 named geraniol 10-hydroxylase (G10H [7]; also renamed G8O; [8]). The third oxidation into the dialdehyde 10-oxogeranial requires a specific alcohol dehydrogenase, 10-hydroxygeraniol oxidoreductase (10HGO, also named 8HGO [9]). Thereafter, iridoid synthase (IS) performs the key step for the assembly of the iridoid heterocyclic ring structure. IS uses 10-oxogeranial and probably couples an initial NAD (P) H-dependent reduction with a subsequent cyclization step to form the ring structure of *cis-trans*-nepetelactol [10]. A second P450 enzyme CYP76A26, 7-deoxyloganetic acid synthase, also named iridoid oxidase (IO), catalyzes a key 3-step oxidation of *cis-trans*-nepetelactol to form 7-deoxyloganetic acid [9, 11]. The latter compound is linked to a glucosyl residue by a substrate specific UDP-glucose glycosyltransferase (UGT), 7-deoxyloganetic acid glucosyltransferase (7DLGT) [9, 12]. The resulting product 7-deoxyloganic acid is hydroxylated at the C-7 position by 7-deoxyloganic acid 7-hydroxylase (7DLH, CYP72A224) [9, 13] to yield loganic acid, which is methylated into loganin by a S-adenosyl-L-methionine: loganic acid methyl transferase (LAMT) [14]. Finally, the ring-opening reaction of loganin in the biosynthesis of secologanin is catalyzed by the fourth P450 of this pathway, secologanin synthase (SLS, CYP72A1) [15]).

Following assembly of the monoterpenoid and indole precursors, formation of the MIA basic skeleton is initiated by strictosidine synthase (STR), which catalyzes the stereospecific condensation of tryptamine with secologanin to form 3α (*S*)-strictosidine [16, 17]. Strictosidine β-D-glucosidase (SGD), catalyzing deglucosylation of strictosidine, produces the last common intermediate in the biosynthesis of the thousand existing MIAs, since the resulting aglycone is the starting point for many different skeletons [18, 19]. The later conversion of the strictosidine aglycone into tabersonine has not been elucidated, but most steps in the final conversion of tabersonine into vindoline have been described. Following 16-methoxylation of tabersonine, performed by the sequential action of tabersonine 16-hydroxylase (T16H) [20-22] and 16-hydroxytabersonine O-methyltransferase (16OMT) [23, 24], 16-methoxytabersonine undergoes an uncharacterized hydration reaction followed by N-methylation, hydroxylation and acetylation carried out by 16-methoxy-2,3-dihydrotabersonine N-methyltransferase (NMT) [25-27], desacetoxyvindoline-4-hydroxylase (D4H) [28, 29] and deacetylvindoline-4-*O*-acetyltransferase (DAT) [30], respectively.

The MIA biosynthetic pathway displays one of the most complex and elaborated forms of compartmentalization described to date (Fig. 1). It was shown to require the coordinated implication of at least four different cell types, implying specific intercellular translocations of metabolite whose identifications are underway. The biosynthesis of MIAs is initiated within internal phloem associated parenchyma (IPAP) cells which host the initial steps leading to secologanin, i.e. the whole MEP pathway together with the eight first reactions of monoterpene-secoiridoid pathway [1, 2, 6, 9-12, 31-33]. The central steps occur in leaf epidermis with conversion of loganic acid into secologanin, after its translocation from IPAP cells. This latter is next conjugated to tryptamine to yield strictosidine, whose corresponding aglycone serves as the primary precursor for complex alkaloids [14, 15, 22, 24, 34-36]. Following translocation of 16-methoxytabersonine or a downstream intermediate, vindoline biosynthesis is completed in laticifers and idioblast cells hosting D4H and DAT activities [34, 37]. In addition, all these biosynthetic steps are marked by a complex subcellular distribution pattern: soluble cytosolic enzymes (TDC, IS, 7DLGT, LAMT, D4H and DAT), endoplasmic reticulum anchored enzymes (G10H, IO, 7DLH, SLS, T16H1 and T16H2), plastidial enzymes (MEP pathway enzymes, GPPS, GES), vacuolar enzymes (STR and PEX1) and nuclear SGD [5, 6, 9, 10, 22, 35-39]. However, despite the existence of multiple intra- and intercellular transports, only one transporter of the MIA pathway has been characterized to date, TPT2, that mediates the specific excretion of catharanthine at the leaf epidermis [40].

Recently, high throughput sequencing approaches (RNA-seq) have been used to provide an access to full *C. roseus* transcriptomes and to help in identifying new genes of the MIA biosynthetic pathway. Such transcriptomes were released by three main initiatives, the Medicinal Plant Genomics Resource (MPGR) [41], Cathacyc and ORCAE [42] (ccOrcae) and Phytometasyn (PMS) [43], as well as other independent studies [44, 45]. These data were generated from the sequencing of libraries prepared from whole-organs and specific experimental conditions. The resulting sequences have been used in orthology and gene clustering allowing the identification of new genes, such as 7DLH and 7DLGT (reviewed in [2]). However, new results have pinpointed the involvement of multiple enzyme isoforms in this highly compartmentalized pathway of MIA biosynthesis, adding thus an additional layer of complexity. Indeed, we have recently described two isoforms of T16H (T16H1 and T16H2), encoded by two distinct genes displaying different tissue-specific expression patterns [22]. However, it should be noted that the currently available *C. roseus* transcriptome resources failed to correctly integrate these isoforms, which could result from improper *de novo* assembly or insufficient sequencing depth of samples. Hence, browsing the current *C. roseus* transcriptome resources might miss important information, highlighting the need for a more exhaustive transcriptome.

Based on this ascertainment, the objective of the present study was to generate a consensus transcriptome containing an exhaustive library of *C. roseus* transcripts with expression level information. Different strategies have been previously employed to generate transcriptome assemblies for non-model animal and plant species. Most of them rely on the combination of assemblies resulting from different assemblers such as Trinity [46], Oases [47], TransAbyss [48] and SOAPdenovo-Trans [49] with eventually different *k*-mer lengths since this criteria is expected to bypass the uneven distribution of transcript abundance [50]. Such strategy was successfully conducted for *Anas platyrhynchos domestica* [51] and *Nicotiana benthamiana* [52], for which assemblies were performed on a unique library, but also for wheat, with assemblies performed on a mix of 4 libraries [53]. In each case, redundancy caused by the merging of different assemblies was decreased by using clustering tools such as CD-HIT-EST [54] or TGICL [55]. In *C. roseus*, a recent study compared assemblies generated by Abyss, Velvet and Oases running with different *k-*mer values on a mix of 3 libraries prepared from different organs and merged the best result with the previous assembly prepared by MPGR [44]. In such a case, mixing samples is expected to increase the possibility to find lowly expressed genes and isoforms, due to the diversity of reads sequenced from diverse tissues/experimental conditions, for instance. However, combining libraries prepared from different sources, such as plant cultivars, may also generate more potential isoforms due to genetic polymorphisms. In the present study, we built a new consensus transcriptome for *C. roseus* using already published data. We generated assemblies for every available sample to take advantage of the diversity of tissues/experimental conditions, combined them and tested different thresholds to cluster homologous contigs. Special attention was taken to reduce the redundancy without affecting transcript quality. Optimization of this consensus assembly was performed by monitoring reconstruction quality of all MIA biosynthetic genes, with a particular emphasis on the two previously described T16H isoforms [22] and on a newly identified SLS isoform whose functional validation is also depicted. The reconstruction of such a *C. roseus* consensus transcriptome is expected to facilitate the identification of the missing MIA biosynthetic enzymes by studying the clustering of gene expression for instance, but also the characterization of new isoforms whose existence could be predicted through this work.

## Results and discussion

### Identification and characterization of a second SLS isoform

**Fig. 2:**
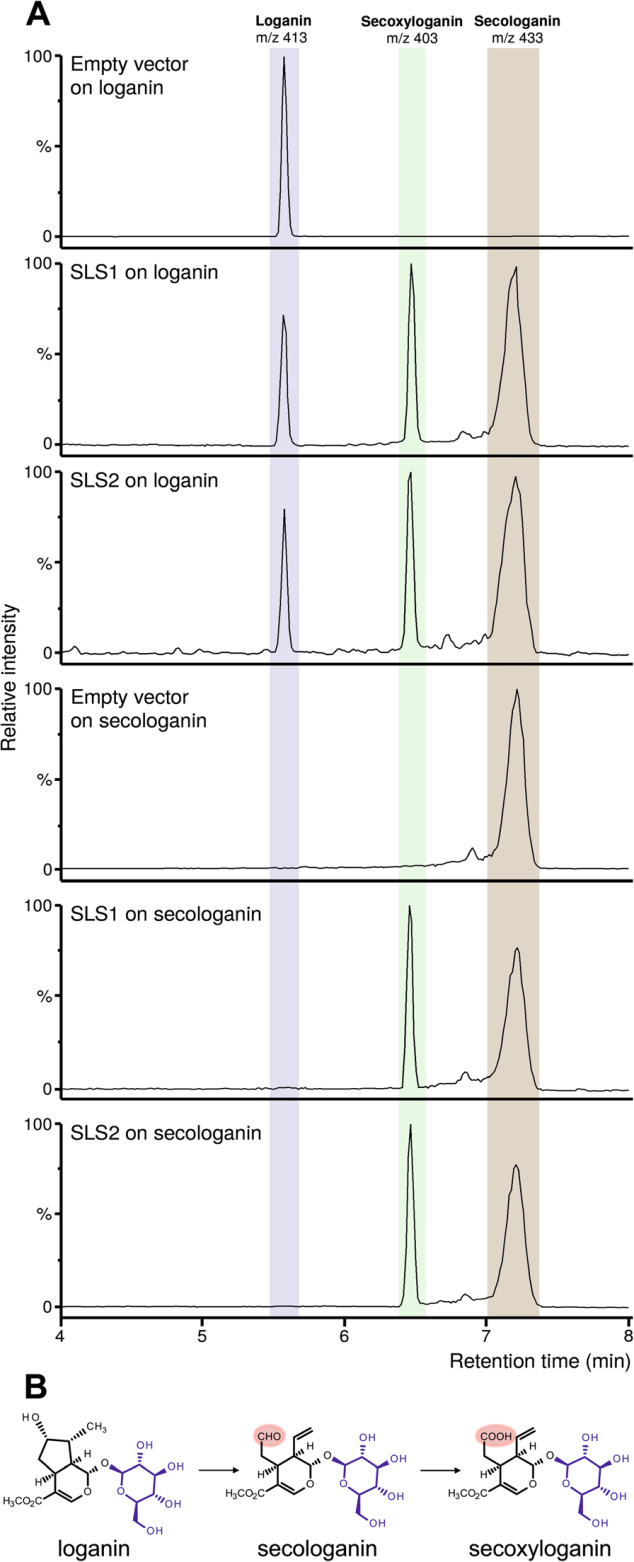
SLS1 and SLS2 catalyze the conversion of loganin in secologanin and secoxyloganin. **a** LC-MS chromatograms using selected ion monitoring (loganin, mass-to-charge ratio 413; secologanin, mass-to-charge ratio 433; secoxyloganin, mass-to-charge ration 403) of the reaction products of yeast extracts from cell cultures expressing either SLS1 or SLS2 or containing the empty pYeDP60 vector, incubated with loganin or secologanin. **b** Schematic reaction catalyzed by SLS1 and SLS2 highlighting the aldehyde to acid conversion

While amplifying the coding sequence of SLS (CYP71A1, Genbank accession number L10081) [14], sequencing of the PCR products revealed the presence of a second putative isoform exhibiting 96 % identity with the original SLS isoform. Interrogation of the *C. roseus* transcriptomic databases (Medicinal Plant Genomics Resource, CathaCyc/Orcae and Phytometasyn) led to the identification of identical but partial sequences confirming thus the existence of this new SLS sequence that has been recently deposited to Genbank under accession number KF415117. The corresponding P450 also displayed a high level of identity (97 %) with the first SLS isoform (Additional file 1: Figure S1) suggesting that it could also catalyze the oxidative ring cleavage of loganin to produce secologanin. To test this hypothesis. The original and the new putative SLS isoforms were individually expressed in the *Saccharomyces* WAT11 strain that overexpresses the Arabidopsis NADPH P450 reductase [56]. Crude extracts of galactose-induced yeasts transformed with the pYeDP60 empty control vector or the pYeDP60 expressing each P450 were subsequently incubated with NADPH, H^+^ and loganin, and analyzed by UPLC-MS (Fig. 2). While no modification of loganin occurred with the empty vector crude extract, a conversion of loganin into secologanin was observed with the crude extract of each enzyme. This established that the putative SLS isoform truly corresponds to a new SLS isoform, named SLS2 as reference to CYP71A1, renamed SLS1.

**Fig. 3:**
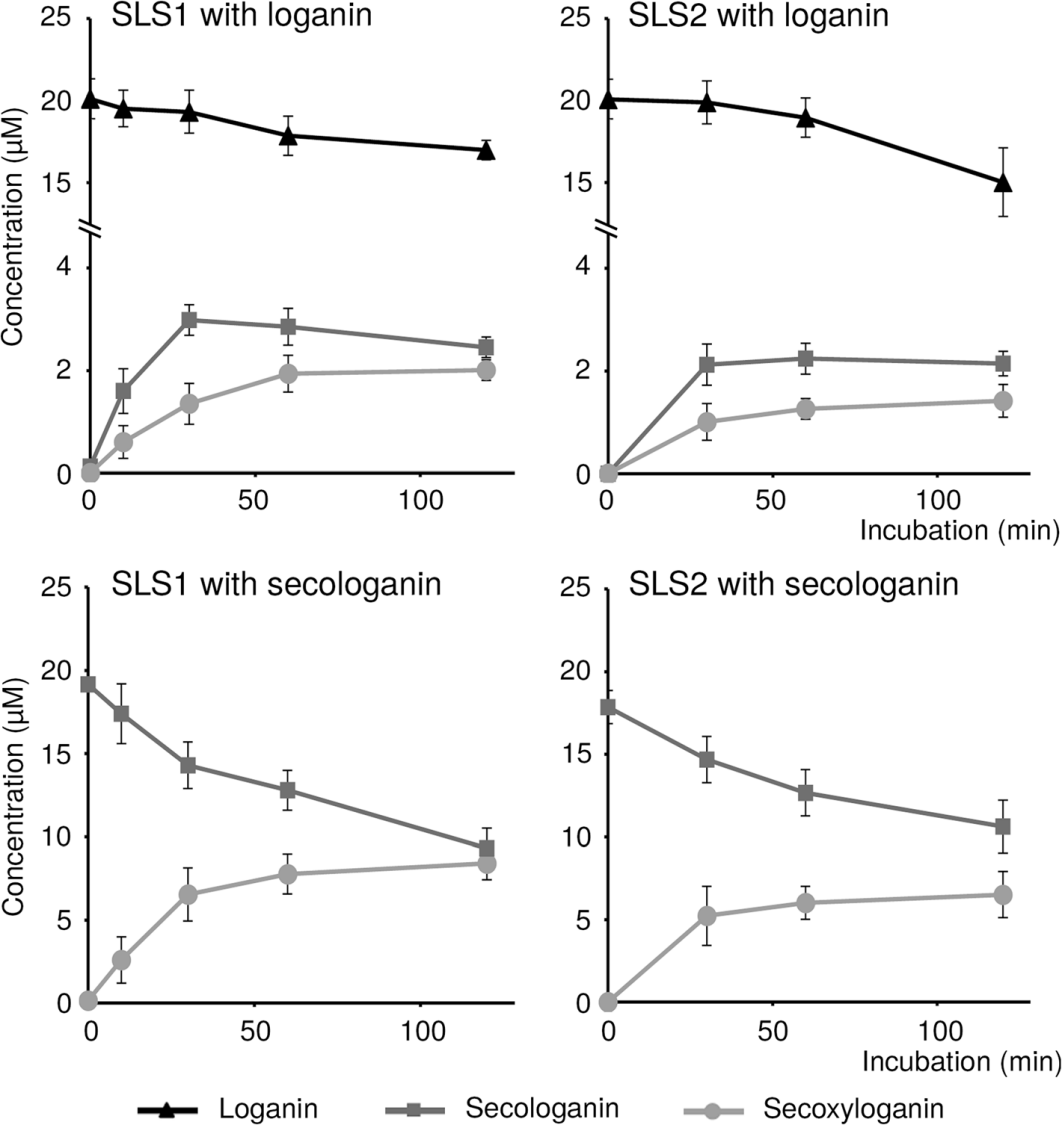
Time course analyses of the reaction catalyzed by SLS1 and SLS2. Proteins extracts from yeast cells expressing either SLS1 or SLS2 were incubated at 30 °C with loganin or secologanin. Formation of the resulting products was monitored by LC-MS analysis. Black triangles, loganin; dark grey squares, secologanin; light circles, secoxyloganin

Interestingly, we noted that both SLS1 and SLS2 also convert loganin into a more polar compound identified as secoxyloganin according to UV and MS spectra and a comparison with a pure authentic standard (Fig. 2; Additional file 2: Figure S2). By contrast, this product was not produced using the empty vector crude extract suggesting that it results from a reaction catalyzed by SLS. For SLS1 and SLS2, time course reactions showed a decrease of the loganin content accompanied by the formation of both secologanin and secoxyloganin (Fig. 3). Since secoxyloganin corresponds to the acidic form of secologanin, it may result from the oxidation of the aldehyde function of secologanin. Therefore, we tested the capacity of both SLS1 and SLS2 to convert secologanin into secoxyloganin. While no formation of secoxyloganin was monitored by incubating secologanin with the empty vector crude extract, both SLS1 and SLS2 directly produce secoxyloganin from secologanin in a stoichiometric manner at least during the early times of the reaction (Fig. 2, Fig. 3). As a consequence, these results suggest that SLS1 and SLS2 not only catalyze the oxidative ring cleavage of loganin to produce secologanin but also perform the oxidation of secologanin into secoxyloganin. Besides G10H and IO, SLS1 and SLS2 constitute the third type of P450 from the seco-iridoid pathway performing more than one catalytic reaction [8, 11]. Interestingly, the additional reaction catalyzed by SLS1 and SLS2 is similar to the third oxidation performed by IO to generate 7-deoxyloganetic acid, suggesting that regiospecific multi-oxidation is rather common to P450s acting in secoiridoid biosynthesis. The occurrence of sequential oxidations has been reported for several P450s [57] but the dissociation of intermediates is still a question of debate since it ranges from an absence of dissociation for P450 11B2 [58] to a dissociation of 85 % for P450 2C11 [59]. In the absence of pulse-chase experiments, we are not able to propose a reaction scheme for both SLS1 and SLS2 concerning secologanin release.

**Fig. 4:**
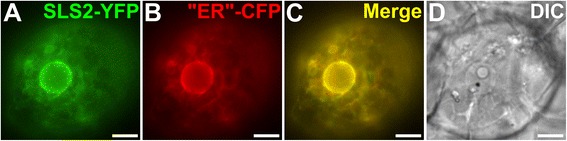
SLS2 is located to the endoplasmic reticulum. *C. roseus* cells were transiently transformed with the SLS2-YFP-expressing vector (SLS2-YFP; (**a**) in combination with the plasmid expressing an ER-CFP marker (“ER”-CFP; (**b**). Colocalization of the two fluorescence signals appears on the merged images (**c**). Cell morphology (**d**) was observed with differential interference contrast (DIC). Bars = 10 μm

While important amounts of secologanin can be measured in the different organs of *C. roseus* [22] we never detected the presence of secoxyloganin (data not shown), raising the question of the physiological signification of this compound production but also suggesting that secologanin could be release from the SLS catalytic site during the sequential oxidation process. Secoxyloganin is an acidic compound derived from secologanin that is no longer able to be condensed with tryptamine by STR in the vacuole, due to the absence of the aldehyde function. If secoxyloganin formation occurs *in vivo*, the resulting depletion of the secologanin pool would be deleterious for the subsequent synthesis of MIAs. Although we cannot exclude that additional enzymes might convert secoxyloganin back to secologanin, the subcellular compartmentation of secologanin biosynthesis may also limit secoxyloganin formation *in planta*. Subcellular localization studies showed that SLS2 is located to the endoplasmic reticulum as previously observed for SLS1 [36] (Fig. 4). Since both SLS1 and SLS2 are anchored to this subcellular compartment and release their product in the cytosol, active transport of secologanin to the vacuolar compartment with high efficiency might allow its import before additional oxidation by SLS1 or SLS2. This would be a direct and interesting consequence of the complex subcellular organization of the MIA biosynthetic pathway regarding regulation of the metabolic flux.

**Fig. 5:**
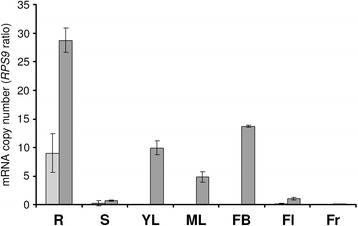
Expression of SLS1 and SLS2 in *C. roseus* organs. SLS1 (light gray bars) and SLS2 (dark gray bars) transcript levels were determined by real-time RT-PCR analyses performed on total RNA extracted from *C. roseus* organs and transcript copy numbers were normalized using CrRPS9. R, Roots; S, stems; YL, young leaves; ML, mature leaves; FB, flower buds; Fl, flowers; Fr, fruits

Besides T16H and IS, SLS corresponds to the third type of enzymes from the MIA biosynthetic pathway displaying more than one isoform [22, 60]. While T16H1 and T16H2 have distinct organ specific-roles in MIA biosynthesis, IS4 and IS5 display somewhat redundant functions. To gain insight into the respective involvement of the two SLS isoforms, SLS1 and SLS2 gene expression was measured in the main *C. roseus* organs (Fig. 5). SLS2 expression was detected in all the tested organs and reached maxima in those directly associated with MIA biosynthesis including roots, flower buds and leaves. By contrast, SLS1 transcripts were barely detectable in all organs except in roots where expression was three-fold lower than SLS2. It is interesting to note that SLS1 was initially characterized from a cell suspension culture cDNA library [15]. Therefore, these results suggest that SLS1 and SLS2 can contribute concomitantly to secologanin biosynthesis in roots while SLS2 can be the prominent isoform of secologanin biosynthesis in the aerial parts of the plant.

### Reconstruction of individual MIA genes in current assemblies and new single assemblies generated with Trinity

Our functional approach clearly demonstrates the existence of MIA enzyme isoforms potentially displaying specific catalytic parameters and/or expression patterns. The identification of new enzyme isoforms is of importance and may notably help in isolating the most efficient enzymes that could be used in synthetic biology approaches as recently highlighted for strictosidine production [61]. RNA-seq data for *C. roseus* provide an important opportunity to retrieve such isoforms by analyzing sequences sharing high identities. In addition, combining gene expression levels from different experimental conditions will improve the quality of transcript expression patterns which allow conducting gene clustering analyses. However, this requires a correct reconstruction of each isoform.

**Fig. 6:**
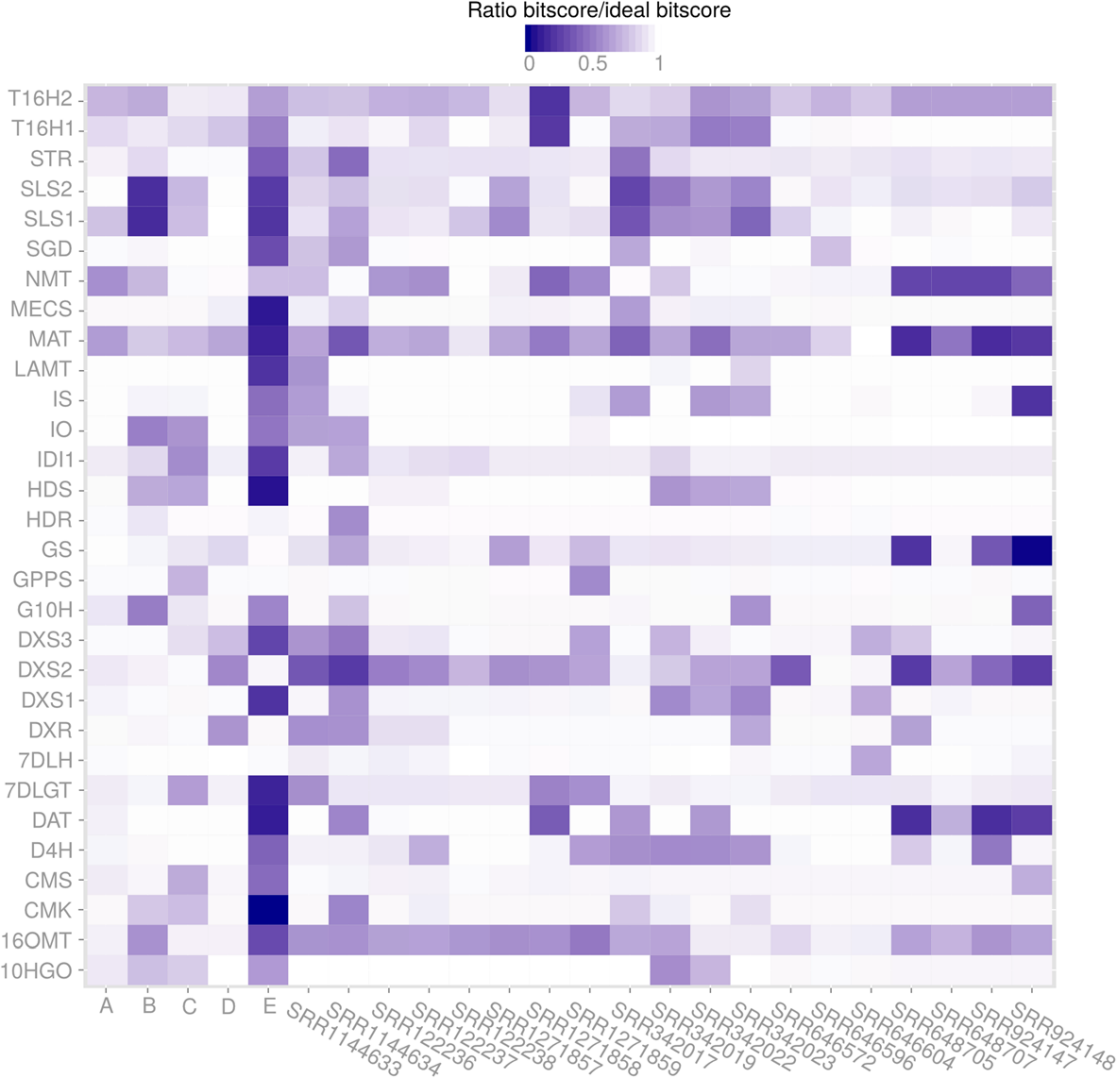
Reconstruction quality of MIA genes in different assemblies. Current resources (A = ccOrcae (Smartcell), B = mpgrCra (Medicinal Plant Genomics Resource), C = NIPGR, D = PMS454 (PhytoMetaSyn), E = PMSIllu (PhytoMetaSyn) and new assemblies (19 SRR with PE sequencing design) were used as databases to identify homologs of MIA genes, and the resulting bitscore obtained by BLAST was compared to that of an ideal sequence (bitscore of the reference sequence against itself, i.e. bitscore ratio = 1)

We performed a detailed inspection of the current transcriptomic resources, available from Medicinal Plant Genomic Resources [41], PhytoMetaSyn [43] (with Illumina reads or with 454 reads), Cathacyc/Orcae [42] and a newly prepared transcriptome by a NIPGR research team [44]. The corresponding datasets will be thereafter named mpgrCra, PMSIllu (Illumina reads), PMS454 (454 reads), ccOrcae and NIPGR, respectively (Additional file 3: Table S1). Our analysis revealed that correct reconstruction of MIA genes was not systematic. Reference sequences of MIA genes available on NCBI were blasted against those assemblies and the bitscore of best hit was compared to that of an ideal reconstruction, i.e. the bitscore of the reference sequence against itself. On the whole, quality of reconstruction was quite unequal between assemblies (Fig. 6). PMS454 and ccOrcae assemblies displayed the best sequences while PMSIllu was of weaker quality (see for example 10HGO, 16OMT, CMK, HDS, IDI1 and IO). NIPGR and mpgrCra assemblies were quite similar in content, probably due to the construction design of the NIPGR assembly (independent libraries assembled and mixed with mpgrCra before filtering). Classically, discrepancies between assemblies might be due to natural polymorphisms, sequencing and/or reconstruction errors. When looking at very well reconstructed genes such as 7DLH and LAMT, it appeared that small differences are related to single-base variations. For 7DLH, such a variation was observed at the position 564 of the reference sequence (KF415115) in the two assemblies mprgCra and NIPGR where a C was changed to A. This variation could be a true SNP (Single Nucleotide Polymorphism) as the reference sequence was obtained with another cultivar (Little Delicata). Concerning isoforms of T16H (T16H1 and T16H2) and SLS (SLS1 and SLS2), it appeared that current assemblies failed to present high quality sequences of the 4 transcripts (T16H1, T16H2, SLS1 and SLS2) simultaneously. For example, while both SLS isoforms were well reconstructed (bitscore/ideal bitscore >0.99) in PMS454, it was not the case for T16H1 (0.78). The best reconstructions of T16H1 and T16H2 were found in mpgrCra (0.92 for T16H1) and NIPGR (0.92 for T16H2), respectively. This result prompted us to try new assembly strategies in order to produce a more complete transcriptome.

To this aim, we next compared new single assemblies of each sample prepared with the Trinity pipeline [46, 62], since the resulting diversity is expected to reveal more MIA biosynthetic genes and isoforms. For this approach, a total of 19 samples were retained because of their paired-end sequencing design as it is expected to improve the quality of reconstruction (Additional file 3: Table S1). Before running Trinity, read content in each sample was normalized by analyzing *k*-mer content (*k*-mer size = 25, maximum coverage = 30) to remove reads being overrepresented or displaying abnormal distribution and their quality was assessed with FastQC (Additional file 4: Table S2). Again, a wide range of reconstruction quality was observed. All MIA biosynthetic genes had a high reconstruction quality (>0.85, minimal highest quality observed T16H2 in SRR1271857 with 0.88) in at least one single assembly. However, as observed for current resources, we did not observe the simultaneous presence of SLS and T16H isoforms, despite the use of mixed libraries (SRR122236, SRR122237 and SRR122238). Interestingly, the base variation within 7DLH described above was no more observable in the alignment of best hits (data not shown) in each single assembly with the 7DLH reference sequence. Hence the variations observed in NIPGR and mpgrCra assemblies probably result from reconstruction errors.

### Preparation of a consensus transcriptome for *C. roseus*

**Fig. 7:**
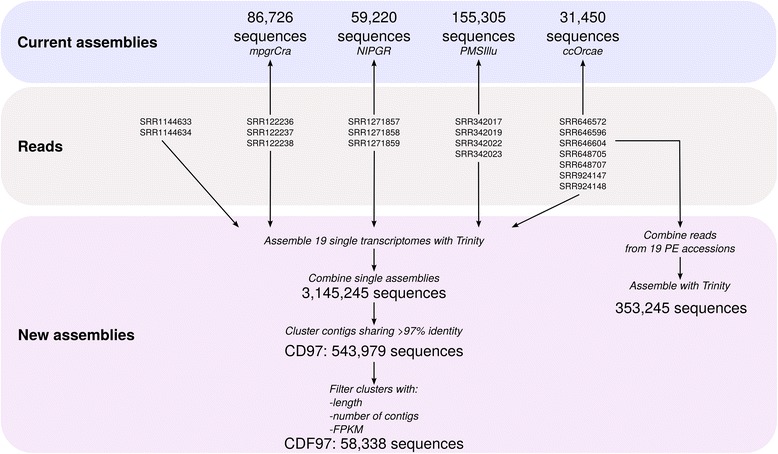
Preparation of a consensus transcriptome using previously published RNA-seq data. Single transcriptomes were *de novo* assembled with Trinity using samples with paired-end design, then combined and similar contigs were subsequently clustered with CD-HIT-EST. Poor quality clusters were removed on the basis of cluster length, composition and expression (FPKM). For trying purposes, we also constructed with Trinity a transcriptome on a dataset including reads from all samples with paired-end design

The quality of reconstruction of MIA biosynthetic genes in single assemblies suggests that raw resources might contain enough information to construct a consensus transcriptome since most of genes displayed a good reconstruction (>0.8) in at least one sample (Fig. 6). Because samples were sequenced at different depth, it may be possible that partial transcripts were also reconstructed in single assemblies. Therefore, two strategies based on the combination of samples were then tested to correctly assemble isoforms and MIA biosynthetic genes (Fig. 7): we first tried to combine all reads and generate a new assembly, while, in the second approach, the individual transcriptomes were combined and the resulting dataset clustered (using CD-HIT-EST) and subsequently filtered (to ensure the removal of clusters with weak representation by reads and in single assemblies).

In the first approach, reads from each paired-end samples were normalized (k-mer size = 25, maximum coverage = 30; Additional file 4: Table S2), combined and the resulting read set was normalized again. The resulting transcriptome contained 353,245 transcripts with a N50 value of 2,036 nt (median 564 nt). This important number of transcripts suggested the presence of fragmented transcripts and CD-HIT-EST was employed with increasing thresholds of sequence identity (90 to 100 %). However, neither this transcriptome nor its corresponding clustered subsets contained high quality sequences (Additional file 5: Figure S3).

**Fig. 8:**
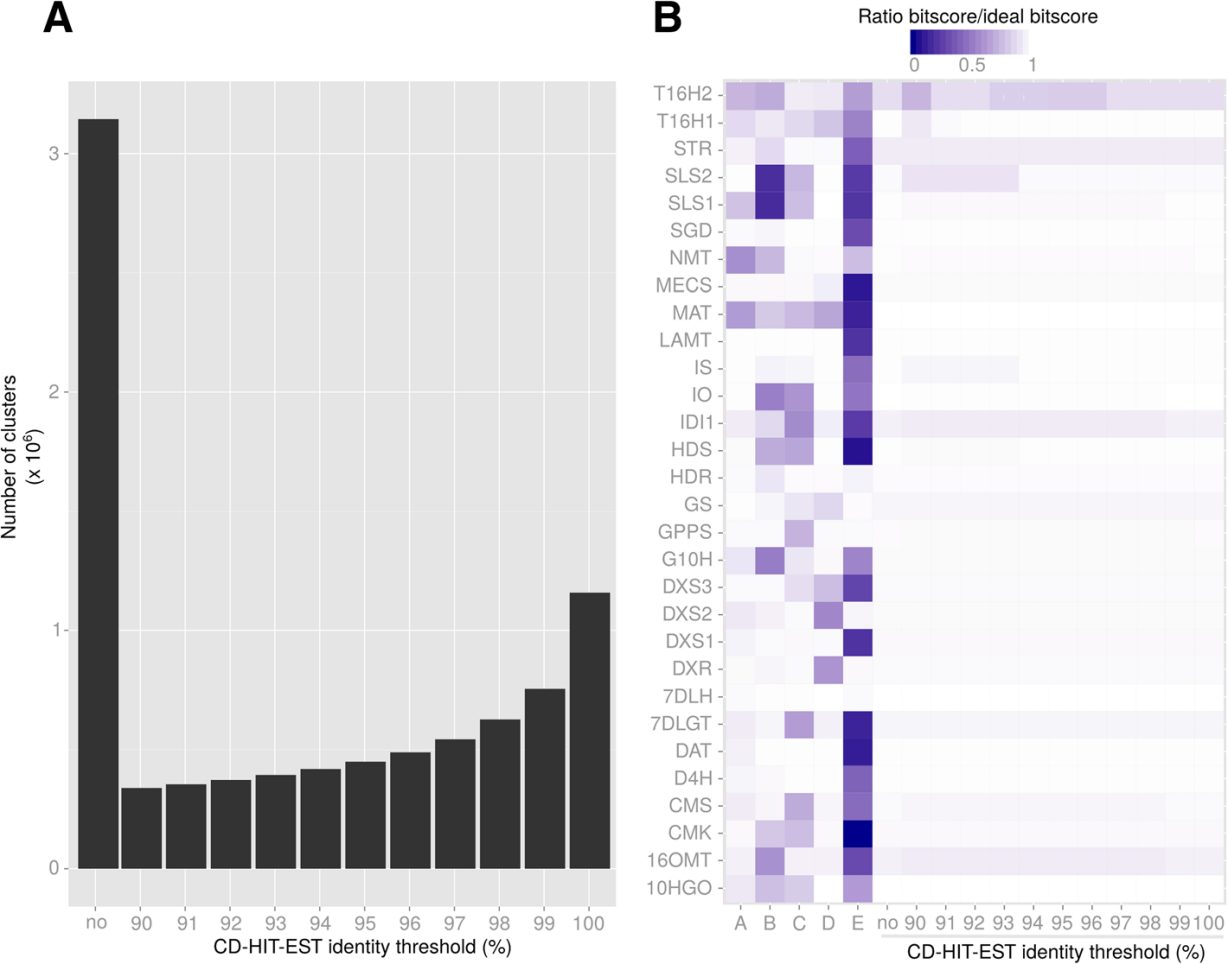
Clustering of redundant contigs in the dataset resulting from the combination of all single assemblies. Contigs sharing a given % of identity were clustered with CD-HIT-EST. **a** Number of clusters after CD-HIT-EST at % identity thresholds fixed from 90 to 100 %. **b** Reconstruction quality of MIA genes in the current resources (A = ccOrcae, B = mpgrCra, C = NIPGR, D = PMS454, E = PMSIllu) and the datasets resulting from the clustering by CD-HIT-EST at % identity thresholds. Reference MIA gene sequences were BLASTed against each assembly and the resulting bitscore was compared to that of an ideal sequence (bitscore of the reference sequence against itself, i.e. bitscore ratio = 1)

In the second approach, we merged all single Trinity assemblies (see above) and ran different filtering procedures in order to decrease the resulting redundancy without altering transcript quality. A total of 3,145,245 contigs from single assemblies were then combined. This allowed combining very high quality transcripts within one new assembly which however, contained an evident redundancy due to the merging procedure. Indeed, the resulting large dataset is expected to cover a large number of isoforms. These isoforms may be real transcripts such as isoforms of SLS and T16H that have to be differentiated, or alleles of different cultivars, which should be integrated into a reference sequence. Running CD-HIT-EST with different sequence identity thresholds succeeded in combining contigs into clusters (Fig. 8a). This algorithm clusters similar sequences and uses one of them as a representative one. A weak decrease in sequence quality was observed with lower identity thresholds for 16OMT (bitscore/ideal bitscore in non-clustered dataset, 0.94; at clustering threshold 98 %, 0.92), IS and SLS2 for clustering thresholds lower than 0.94 (Fig. 8b). The transcript with lowest quality was T16H2 (0.88 for clustering threshold above 0.96). However, its quality was quite similar with that of the best reconstruction in current resources (0.92 in NIPGR). Two other genes, IDI1 and STR did not display ideal reconstruction, according to the reference sequence. IDI1 was slightly better reconstructed in PMS454 and STR was better in NIPGR and PMS454. The origin of those discrepancies are unclear but might have been caused by a higher polymorphism, leading to a different reference sequence in comparison to the representative clusters obtained here. According to the quality of MIA biosynthetic gene reconstruction, we further retained the clustered dataset obtained with a sequence identity threshold of 97 %. This threshold should be permissive enough to combine alleles differing by only few SNPs. The resulting clustered dataset, thereafter renamed CD97, was composed of a total of 534,979 clusters, 357,652 being singletons (a contig displaying no sufficient identity with other contigs) and 177,327 being real clusters, containing more than two contigs (which may originate from the same single assembly or from different single assemblies). A total of 249,423 sequences had identities (e-value < 1e-20) with sequences of the Uniprot database (Blastx), and 9,283 proteins found in this database were represented at 90 % of their length by at least one cluster in CD97.

**Fig. 9:**
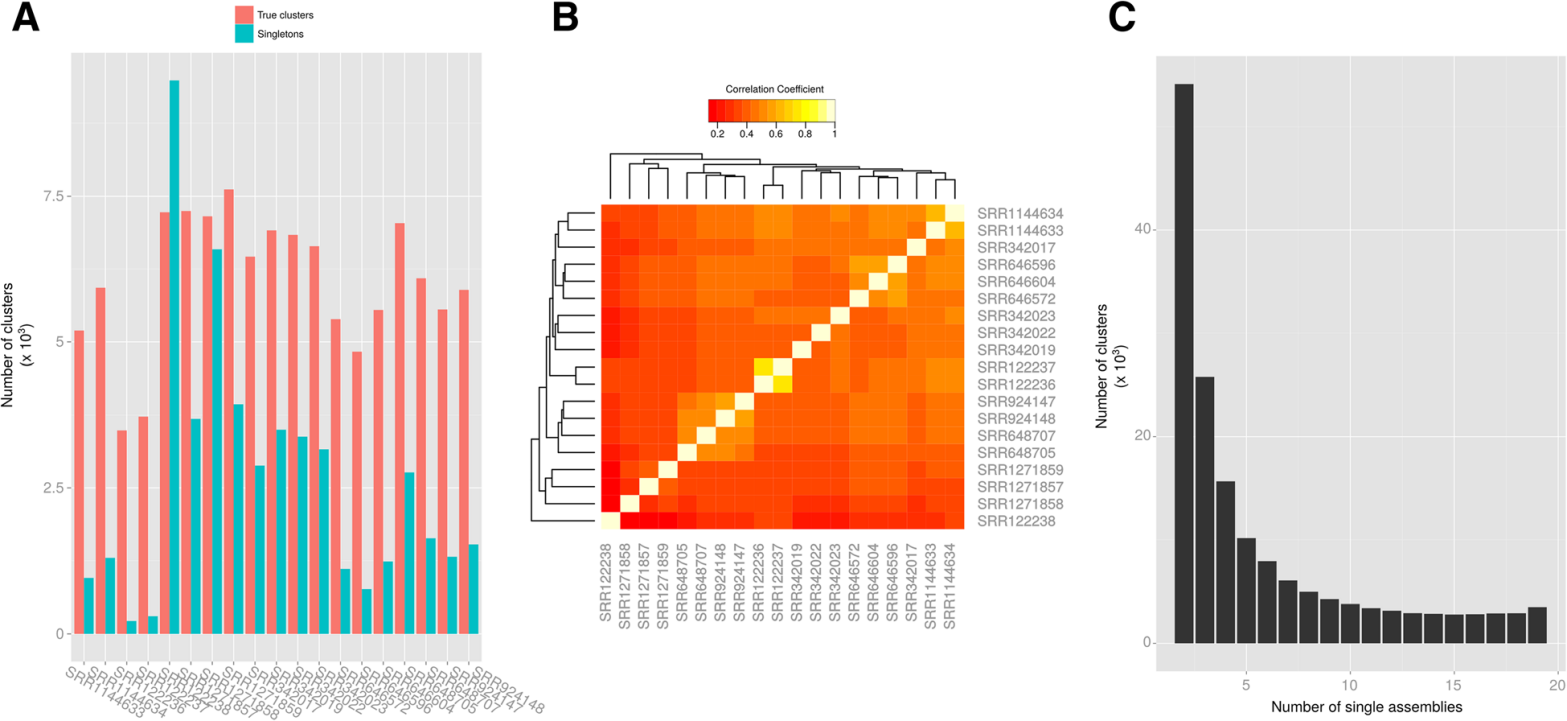
Composition of the clustered dataset (CD97) resulting from the processing of the combination of all single assemblies with CD-HIT-EST at 97 % identity. **a** Integration of contigs from single assemblies into clusters. Contigs which cannot be grouped with others are called singletons. True clusters, i.e. containing at least two different contigs, may have been formed by the combination of contigs from one or more initial single assemblies. **b** Correlation plot of single assemblies. Contigs found in each cluster (singletons and true clusters) were identified and counted per initial assembly. Two initial assemblies are therefore strongly correlated (Pearson Correlation Coefficient) if their contigs are found in the same clusters. **c** Composition of true clusters. This graph shows how many single assemblies are represented within clusters

Participation of initial single assemblies in CD97 clusters was homogeneous, except for SRR122238 for which only 10 % of contigs (4.3 % in clusters, 5.7 % in singletons; Additional file 4: Table S2) were used by CD-HIT-EST. Concerning SRR122238 single assembly, the low proportion of reads used in CD97 was probably due to its very high number of contigs (1,666,984) in comparison with the other assemblies. For other single assemblies, more than 90 % of contigs were used by CD-HIT-EST, with at least 50 % in true clusters (Fig. 9a; Additional file 4: Table S2). Composition of true clusters revealed a somewhat preferential association of contigs from single assemblies obtained in a same study (Fig. 9b). Correlation coefficients calculated on the pattern of participation of each single assembly in true clusters were higher for 4 groups of samples: (i) SRR1144633 and SRR1144634 (SRP035766, leafy flower transition study), (ii) SRR646596, SRR646604 and SRR646572 (SRP017832, MeJA treatments on shoots), (iii) SRR122237 and SRR122236 (SRP005953, mixed libraries from different organs) and (iv) SRR924147, SRR924148, SRR648707 and SRR648705 (SRP026417 and SRP017947, cell suspension MeJA and ORCA overexpression). This preferential association is more likely to be due to the inherent genetic diversity between *C. roseus* cultivars than experimental conditions. However, high coefficient correlations (>0.6) were also observed for independent studies, as exemplified between SRR122236 and SRR1144634. The strongest differences were observed for samples of the NIPGR study (SRR1271857, SRR1271858 and SRR1271859) and for SRR122238. For the latter, this might be linked to its higher participation in singletons than in true clusters (Fig. 9a). In CD97, 105,730 clusters contained contigs from 2 to 5 different single assemblies, 31,055 clusters contained contigs from more than 10 single assemblies and 3,506 clusters were composed of contigs from the 19 single assemblies (Fig. 9c). These 31,055 clusters might represent the core transcriptome of *C. roseus*. Indeed, 25,692 had significant (e-value < 1e-20) identities with proteins of the UniprotKB database (Blastx) (Table 1).

**Table 1:** Transcript annotation and analysis of full-length transcript reconstruction

Assembly	ccOrcae	mpgrCra	NIPGR	PMS454	PMSIllu	CD97	CD97 best clusters^c^	CDF97
Number of transcripts	31,450	86,726	59,220	26,804	155,305	543,979	31,055	58,338
Total annotated transcripts^a^	16,727	55,073	24,142	15,868	22,716	249,413	25,692	49,128
% Annotated transcripts^a^	53.19	63.50	40.77	59.20	14.63	45.85	82.73	84.21
	Cumulative number of proteins^b^							
% Length coverage								
100	4,539	4,115	3,540	1,546	3,168	7,155	5,006	5,734
90	5,686	5,547	5,124	2,127	4,125	9,283	6,198	7,110
80	6,370	6,626	6,377	2,624	4,783	10,903	6,913	7,926
70	6,855	7,641	7,469	3,193	5,381	12,410	7,466	8,572
60	7,298	8,703	8,372	3,827	5,940	14,048	7,958	9,135
50	7,724	9,670	9,119	4,575	6,574	16,085	8,350	9,640
40	8,102	10,528	9,686	5,457	7,177	18,610	8,671	10,033
30	8,479	11,282	10,141	6,497	7,793	21,831	8,908	10,489
20	8,767	11,744	10,393	7,367	8,303	24,619	9,021	10,520
10	8,816	11,830	10,438	7,588	8,430	25,229	9,044	11,137

^a^,Blastx analysis vs UniprotKB/Swiss-Prot. A transcript was considered to be annotated if it matches a protein in the database at a e-value threshold of 1e-20

^b^,Reports the cumulative number of proteins in UniprotKB/Swiss-Prot matched by at least one transcript in the corresponding assembly at a given % coverage

^c^,Clusters in CD97 having contigs from at least ten different single assemblies

**Fig. 10:**
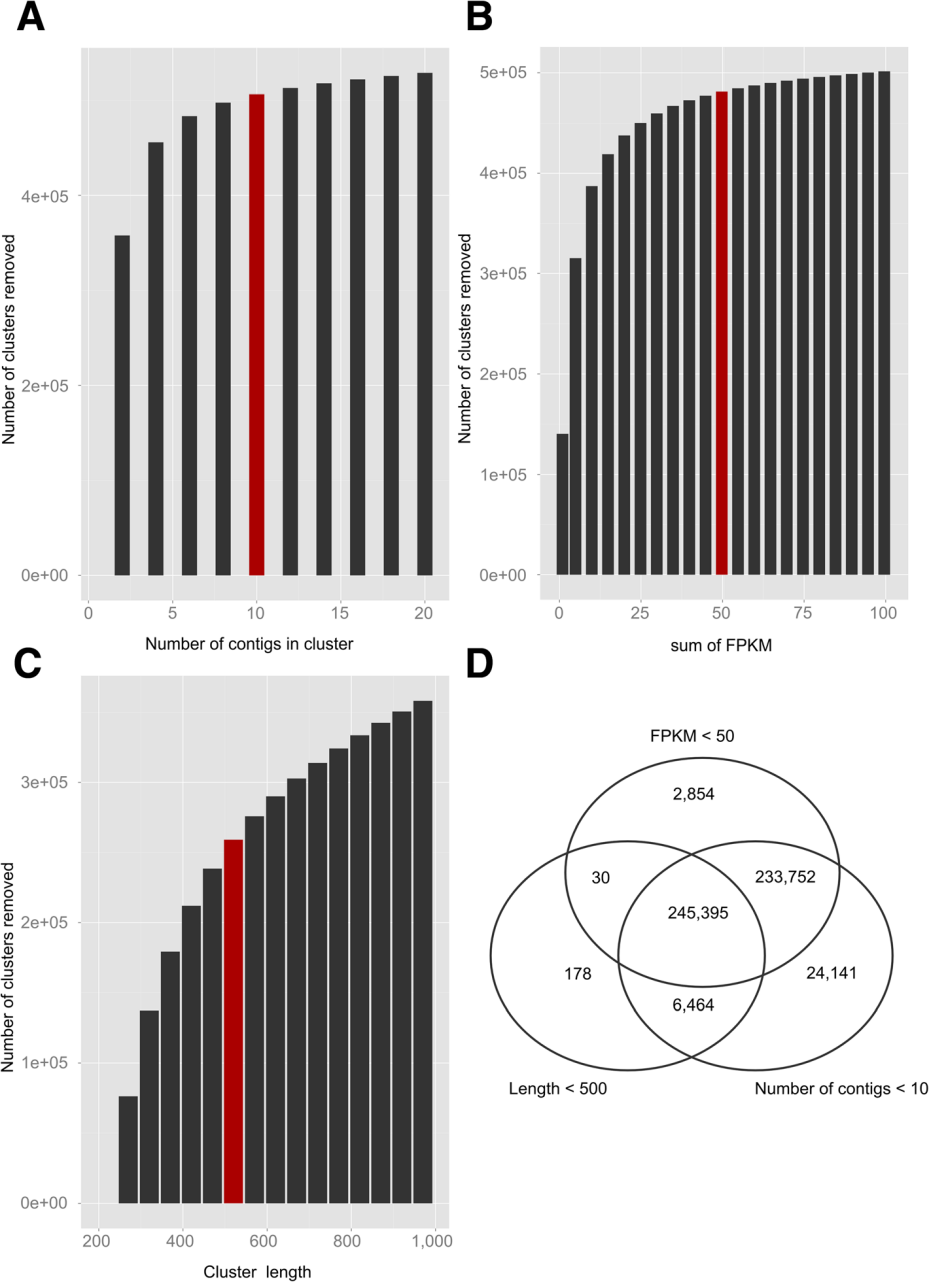
Characterization of clusters in the clustered dataset (CD97). These graphs represent the number of clusters falling below different threshold values for the number of contigs (**a**), the summed FPKM (on the 42 available samples) (**b**) and cluster length (**c**). Red bars show the thresholds that were retained to filter poorly supported sequences from CD97. Sequences which met at least two of those criteria were discarded (**d**) (total of 485,641)

To further clean CD97, all putative clusters were tested for 3 criteria: (i) length, (ii) number of contigs and (iii) expression level (sum of Fragment per Kilobase per Million of reads (FPKM) calculated on the 42 samples (19 paired-end and 23 single-end, see Additional file 3: Table S1). Visual inspection of the number of clusters potentially removed by each filter (Fig. 10) was used to choose appropriate values. The objective was to eliminate clusters with poor representation which could be reconstruction artefacts. Choosing low thresholds of number of contigs and FPKM quickly removed a high number of clusters (427,494 with less than 3 contigs and 315,357 with sum FPKM < 5; Fig. 10a and b). For these two filters, we choose to retain values at which changes in the number of removed clusters displayed lower variation:10 contigs per cluster and sum of FPKM >50. Concerning cluster length, the distribution was more graduated (Fig. 10c). In order to avoid removing weakly expressed or small genes, we chose to discard clusters that do not meet at least two of the three filters (Fig. 10d). We expected that this procedure could reduce the loss of weakly expressed genes or rare isoforms. By fixing a minimal length of 500 bp, a number of contigs > 10 and a sum of FPKM > 50, we found that a large number of sequences (245,395) did not pass the three filters. This indicated that many clusters which size was < 500 bp have both poor representation and weak expression levels. We also found 233,752 clusters which had a sum of FPKM < 50 and contained less than 10 contigs. All sequences having a null sum of FPKM fell in this class. Out of the 543,979 clusters of CD97, a total of 485,641 sequences did not pass the filters. The resulting dataset, which contained 58,338 clusters, was retained and called CDF97.

Out of these 58,338 clusters in CDF97, 49,128 sequences had a significant (e-value < 1e-20) identity within the UniprotKB database (Blastx) (Table 1). Our filtering process obviously decreased the number of potentially annotated proteins (249,423 sequences in CD97), and to a lower extent the number of full-length proteins in Uniprot represented by more 90 % of their length (9,283 in CD97, 7,110 in CDF97). However, given the large number of clusters that were removed, we considered that this loss was limited. Very rare isoforms may have been discarded in CDF97 by the filtering procedure but only if they had weak single assembly representation and low expression level. In addition, the number of full-length proteins in CDF97 was still higher than in the previously published datasets (Table 1). Looking for such isoforms will therefore require a more detailed examination of CD97 (non-filtered dataset). The total number of clusters in CDF97 was higher than other assemblies (31,450 in ccOrcae and 26,804 in PMS454) but similar to that of 59,220 in NIPGR and lower than that of mpgrCra (86,726). Therefore, it is likely that all redundancy has not been removed in CDF97. As a consequence, detailed inspection of expression levels together with functional studies will be required to further clean CDF97.

### Validation of the consensus FPKM-filtered-CDHIT-94 transcriptome through transcript abundance estimation

**Fig. 11:**
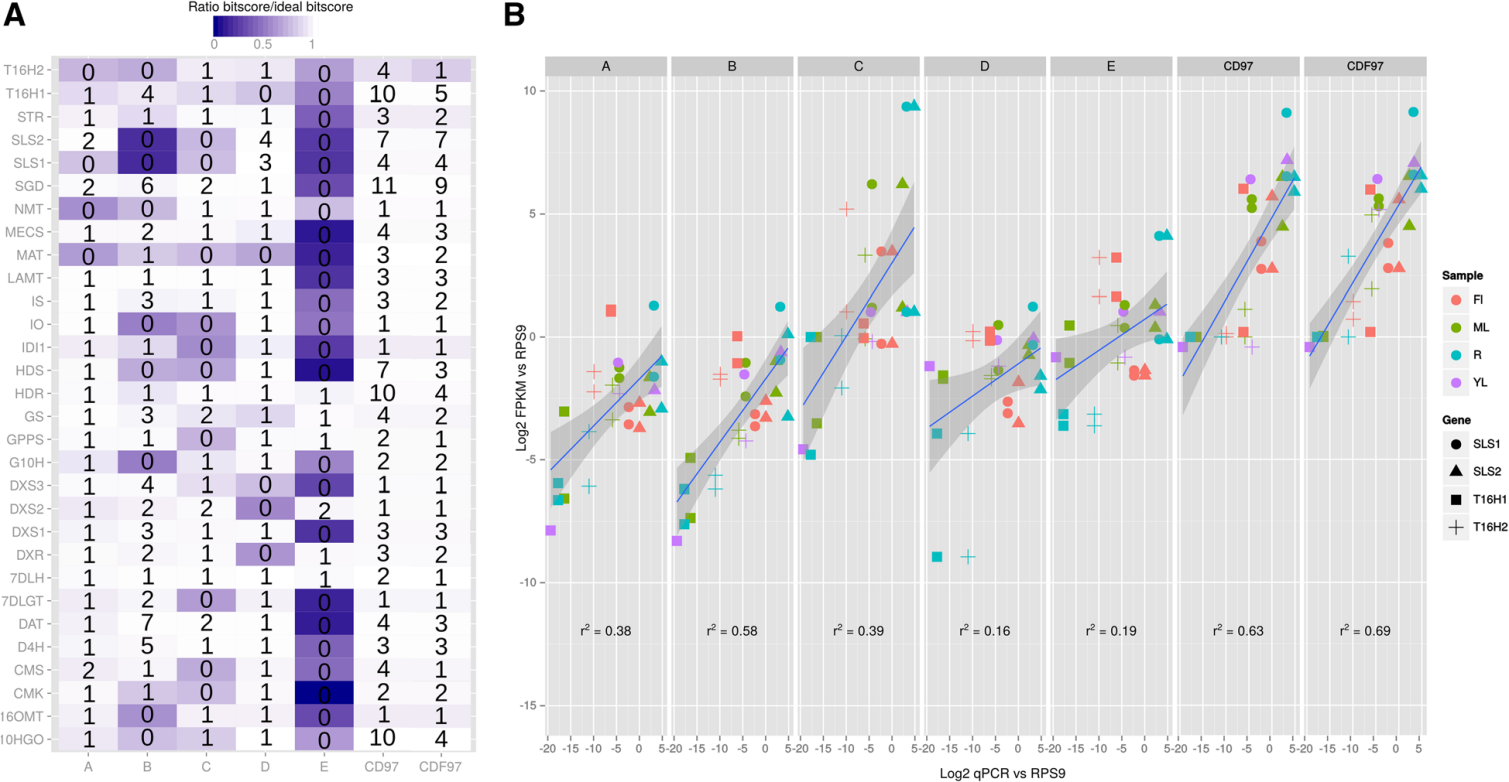
CDF97 performance in quality reconstruction and transcript expression level. **a** Current resources (A = ccOrcae, B = mpgrCra, C = NIPGR, D = PMS454, E = PMSIllu), CD97 and CDF97 were used as databases to identify homologs of MIA genes, and the resulting bitscore obtained by BLAST was compared to that of an ideal sequence (bitscore of the reference sequence against itself, i.e. bitscore ratio = 1). Values inside each cell indicate the number of hits having a ratio >0.8. **b** Comparison of log2 expression levels measured by qPCR and RNA-seq for SLS and T16H isoforms in current assemblies, CD97 and CDF97. RNA-seq samples used for this comparison were: flowers (Fl, SRR122239 and SRR1271859), mature leaves (ML, SRR122251 and SRR1271857), roots (R, SRR122254 and SRR1271858) and young leaves (YL, SRR122252). Expression levels were normalized by RPS9, in both qPCR and RNA-seq measurements. The linear regression line is shown in blue with shading corresponding to 95 % confidence intervals. The r² values indicate correlation coefficients of the linear models

The gene expression levels of SLS and T16H isoforms were determined by qPCR and compared to FPKM values calculated according to the RSEM procedure for each transcript within each assembly. As each isoform has apparent specific expression patterns (Fig. 11b; [22]), the correct alignment of reads to high quality sequences should be able to yield similar results.

More than 1.1 billion reads from the 42 *C. roseus* samples were mapped back with Bowtie2 [63] to current assemblies, as well as our datasets CD97 and CDF97. Substantial differences were observed in the total number of correctly aligned reads between assemblies: 81.69 % on ccOrcae, 61.75 % on mpgrCra, 73.54 % on NIPGR, 61.75 % on PMS454, 53.54 % on PMSIllu, 90.32 % on CD97 and 89.01 % on CDF97. To estimate abundance of clusters in CD97 and CDF97, a contig to cluster map was prepared, similarly to the procedure used in RSEM relying on a transcript to gene map. This was expected to ensure a correct estimation of expression, in particular for rare isoforms (e.g. found in only one sample) displaying slight sequence difference from the cluster representative sequence. Best hits for SLS and T16H isoforms were selected within each assembly. SLS1, SLS2, T16H1 and T16H2 sequences were respectively: Caros007144, Caros020659, Caros001600 and Caros025399 in ccOrcae; cra_locus_10318_iso_2_len_357_ver_3, cra_locus_1389_iso_1_len_312_ver_3, cra_locus_6184_iso_8_len_1687_ver_3 and cra_locus_6184_iso_10_len_1687_ver_3 in mpgrCra; Cr_TC01142 (SLS1 and SLS2), Cr_TC26727 and Cr_TC35206 in NIPGR; CROWL1VD_rep_c387, CROWL1VD_rep_c782, CROWL1VD_rep_c1347 (T16H1 and T16H2) in PMS454; cro. CRO1L1VD_velvet--Contig19748 (SLS1 and SLS2) and cro. CRO1L1VD_velvet--Contig11543 (T16H1and T16H2) in PMSIllu; SRR648707|TR19558|c0_g2_i4, SRR646572|TR30446|c0_g1_i1, SRR648707|TR1325|c0_g1_i2 and SRR1271857|TR29335|c0_g2_i12 in CD97; SRR648707|TR19558|c0_g2_i4, SRR646572|TR30446|c0_g1_i1, SRR648707|TR1325|c0_g1_i2 and SRR342019|TR37243|c0_g2_i1 in CDF97. Interestingly, the best hit for T16H2 differed between CD97 and CDF97. This indicated that the best form in CD97 was either not sufficiently expressed or not represented in the single assemblies to be conserved in CDF97. Hence in CDF97, a slightly less similar sequence for T16H2 was retained albeit being better represented in reads and assemblies (Fig. 11a). Expression levels were calculated as FPKM and normalized to the best hit of *C. roseus* RPS9 gene sequence (Caros004092, for ccOrcae; cra_locus_1407_iso_8_len_924_ver_3 for mpgrCra; Cr_TC15537 for NIPGR; CROWL1VD_rep_c282 for PMS454; cro. CRO1L1VD_velvet--Contig5697 for PMSIllu; SRR646572|TR4777|c0_g1_i1 for CD97 and CDF97).

The expression of each isoform was monitored in immature leaves, mature leaves, flowers and roots. Despites the use of different matrices for FPKM and qPCR analyses, interesting results were observed by comparing both types of measurement. Using current assemblies, we found that expression levels measured on mpgrCra displayed the best correlation with qPCR measurements (linear regression, r^2^ = 0.58; Fig. 11b). For this assembly, T16H2 expression was mixed with that of T16H1 since FPKM indicated similar expressions in flower and immature leaves, while we previously showed by qPCR that T16H1 accumulated in flowers but not T16H2 [22]. The highest correlation was observed for CDF97 (r^2^ = 0.69; Fig. 11b). The contig-to-cluster map used for CD97 and CDF97 (similar to the transcript to gene procedure in RSEM) apparently allowed a more precise calculation of cluster expression values. The higher correlation coefficient obtained with CD97 and CDF97 were probably due to this procedure which was expected to encompass slight polymorphisms that would have impeded read alignment on representative sequences of clusters. Because both the contig and representative sequence (which came from a single assembly) belong to the same cluster, expression levels are calculated for each contig and subsequently attributed to the representative sequence. Contigs specifically expressed in one given sample, having punctuate sequence variation (e.g., due to genetic diversity, but corresponding to the same entity) are thereby used to estimate the expression level of the representative sequence in this sample. Taken together, these results are good indicators of the validity of our CDF97dataset, in both sequence reconstruction and expression levels.

### Exploitation of CDF97 transcriptome for prediction of other MIA biosynthetic gene isoforms

Our finding that gene isoforms may encode similar enzymes (SLS, this study; T16H [22]) potentially add another layer of complexity to the MIA biosynthetic pathway. To predict putative new isoforms, we therefore looked at the number of hits having a score ratio > 0.8 within each assembly. This threshold was empirically determined as lower enough to reveal potential isoforms. According to Fig. 11a, SLS isoforms are identified in PMS454, CD97 and CDF97, as indicated by the presence of more than 1 hit for the corresponding gene. Similarly, isoforms of T16H are observable in NIPGR, CD97 and CDF97. It also appeared that our dataset CDF97 still displayed redundancy among sequences as the number of hits for some genes was still important (10HGO, HDR, HDS, SGD and SLS2). Rather than being true isoforms, it is probable that our clustering and filtering procedures were not stringent enough to remove all redundant sequences. However, in our approach, a special attention was given to avoid discarding rare isoforms. Therefore, genes having 2 or 3 hits with a score ratio > 0.8 (e.g., LAMT or G10H) would merit further studies to determine whether they correspond to true isoforms (separate genes or alternative transcripts) or only simple alleles of different cultivars. For instance, two similar sequences were predicted for IS in CDF97, which correspond to the recently reported IS homologs in *C. roseus* [60].

## Conclusions

Besides T16H and IS, the identification of a second SLS isoform shade light anew on the existence of multiple isoforms of MIA biosynthetic enzymes in *C. roseus*. Apart from the complex cellular and subcellular organisation of the MIA pathway, such a potential enzyme multiplicity constitutes another element implemented along evolution to ensure an efficient and modular production of MIA. It also raises interesting questions regarding the regulation of the MIA metabolic fluxes as suggested by the capacity of both SLS1 and SLS2 to produce secologanin and secoxyloganin, but also regarding evolution of P450s from the seco-iridoid pathway that catalyze more than one reaction.

All these questions strengthened the necessity to develop new tools facilitating the identification of MIA biosynthetic enzymes as well as their potential isoforms. Our reconstructed assembly constitutes thus one of the most optimized transcriptomic resources for *C. roseus* that will facilitate future identification of homologs in the MIA biosynthetic pathway enzymes as well the discovery of uncharacterized enzymes through analyses of gene expression correlation as recently described [1, 2] and demonstrated [64, 65]. This resource opens new perspectives toward the understanding of the whole MIA biosynthetic pathway and remains complementary to genomic sequence analysis.

## Methods

### Heterologous expression of SLS1 and SLS2 in yeast

Full length SLS1 and SLS2 cDNA were amplified using the pair of primers SLS1for (CTGAGAAGATCTATGGAGATGGATATGGATACCATTAG)/SLS1rev (CTGAGAAGATCTCTAGCTCTCAAGCTTCTTGTAGATG) and SLS2-pYEfor (CTGAGAAGATCTATGGAGATGGATATGGATATCATTAGAAAG)/SLS2-pYErev (CTGAGAAGATCTTTAAAAATTCTGTCTCTCAAGCTTCTTGTAGATA), respectively. Both primer couples include *Bgl*II restriction sites at both extremities to allow cloning of the resulting PCR product in the *Bam*HI site of pYeDP60. Both recombinant plasmids and the empty plasmid were independently used to transform the *S. cerevisiae* strain WAT11 expressing the *A. thaliana* NADPH P450 reductase 1 [56]. Yeasts were grown in 10 ml of CSM medium (Yeast Nitrogen Base 0.67 %, dextrose 2 %, drop-out mix without adenine and uracil 0.05 %) until reaching the stationary phase of culture and prior being harvested by centrifugation. Protein expression was induced by cultivating the harvested yeast in 50 ml of YPGal medium (1 % bacto peptone, 1 % yeast extract, and 2 % Galactose) for 6 h as described in [22].

### Enzyme assays

Following induction of protein expression, 50 mL of yeast culture were harvested by centrifugation and resuspended in 2 mL of buffer R (Tris–HCl pH7.5, 50 mM; EDTA 1 mM) in a 50 ml centrifugation tube. An equal volume of glass beads were added (425–600 μm, Sigma) and cells were broken by vigorous shaking. Briefly, tubes were shaked by hand during 30 s in a cold room (4 °C) before being put on ice for 30 additional seconds. This operation was repeated ten times before the addition of two volumes of buffer R allowing the recovering of the yeast crude extracts prior to protein quantification using the Bio-Rad protein microassay. SLS1 and SLS2 activities were analyzed in a final volume of 100 μl containing 600 μg of proteins, 200 μM of NADPH,H^+^ and either 20 μM of loganin or secologanin. Reactions were initiated by addition of NADPH,H^+^, incubated at 30 °C during 10, 30, 60 or 120 min and quenched by addition of 100 μl of methanol prior to ultra-performance liquid chromatography-mass spectrometry analysis (UPLC-MS).

### UPLC-MS analyses

All samples were centrifuged and the supernatants were stored at 4 °C prior to injection. UPLC chromatography system consisted in an ACQUITY UPLC (Waters, Milford, MA, USA). Separation was performed using a Waters Acquity HSS T3 C18 column (150 mm × 2.1 mm, 1.8 μm) with a flow rate of 0.4 mL/min at 55 °C. The injection volume was 5 μL. The mobile phase consisted of solvent A (0.1 % formic acid in water) and solvent B (0.1 % formic acid in acetonitrile). Chromatographic separation was achieved using an 8-min linear gradient from 10 to 24 % solvent B. MS detection was performed by using a SQD mass spectrometer equipped with an electrospray ionization (ESI) source controlled by Masslynx 4.1 software (Waters, Milford, MA). The capillary and sample cone voltages were 3,000 V and 30 V, respectively. The cone and desolvation gas flow rates were 60 and 800 Lh^−1^. Data collection was carried out in negative mode for secoxyloganin ([M-H]^−^ = 403, RT = 6.42 min) and secologanin ([M + HCOOH*-*H]^−^ = 433, RT = 7.12 min) and in positive mode for loganin ([M + Na]^+^ = 413, RT = 5.61 min). Standard calibration curves for secoxyloganin, secologanin and loganin were prepared (1–25 μM) with known pure standards from Chemtek (Worcester, MA, USA), Phytoconsult (Leiden, The Netherlands) and Extrasynthese (Genay, France), respectively.

### Subcellular localization of SLS2

The subcellular localization of SLS2 was determined according to the procedures described in [39]. Briefly, the SLS2 coding sequence was amplified by PCR using primers SLS2-YFP-for (CTGAGAACTAGTATGGAGATGGATATGGATATCATTAGAAAG) and SLS2-YFP-rev (CTGAGAACTAGTAAAATTCTGTCTCTCAAGCTTCTTGTAGATA) and cloned into the *Spe*I restriction sites pSC-A cassette YFPi plasmid in frame with the 5′ extremity of the YFP coding sequence. The resulting plasmid was used for transient transformation of *C. roseus* cells by particle bombardment in combination with a plasmid expressing the ER-CFP marker [66].

### Gene expression analysis

SLS1 (L10081) and SLS2 (KF415117) expression was measured by real-time RT-PCR using primers SLS1_QPCR1-for (TAAACCTGAGTTTGAACGCTTAAATCAC)/SLS1-QPCR1-rev (GACAATCTTTGTTAGATCAATCACTGGT) and SLS2_QPCR1-for (CAAGCCTGAATTTGAACGCTTGAATCAT) and SLS2_QPCR1-rev (AATAATCTTGGTCAGATCAATAACTGGC). PCR products were cloned in pGEM-Teasy according to the manufacturer protocol and Sanger sequenced to ensure the specificity of amplification. Primer efficacity and cross-amplification was tested on plasmids containing either SLS1 or SLS2 coding sequence. Different *C. roseus* organs (such as roots, stems, young and mature leaves, flower buds, flowers, and fruits – Apricot sunstorm cultivar) were immediately frozen in liquid nitrogen after sampling. Samples (50 mg) were ground with a mortar and a pestle in liquid nitrogen and total RNA were extracted with the RNeasy Plant mini kit (Qiagen), controlled with a Nanodrop spectrophotometer (ThermoFisher) and treated (1.5 μg) with RQ1 RNase-free DNase (Promega) before being used for first-strand cDNA synthesis by priming with oligo (dT) 18 (0.5 μM). Retro-transcription (RT) of 1.5 μg of total RNA was carried out using the SuperScript III reverse transcriptase kit (Invitrogen) at 50 °C during 1 h according to manufacturer’s instructions. Real-time PCR was run on a CFX96 Touch Real- Time PCR System (Bio-Rad) using the SYBR Green I technology. Each reaction was performed in a total reaction volume of 25 μL containing an equal amount of cDNAs (1/3 dilution), 0.05 μM forward and reverse primers, and 1 × DyNAmo™ ColorFlash Probe qPCR Kit (Termo Fisher Scientific). The amplication program was 95 °C for 7 min (polymerase heat activation), followed by 40 cycles containing 2 steps, 95 °C for 10 sec and 60 °C for 40 sec. At the end of the amplification, a melt curve was performed to check amplification specificity. Absolute quantification of transcript copy number was performed with calibration curves and normalization with the *C. roseus* 40S Ribosomal protein S9 (RPS9 – primers qRPS9for TTACAAGTCCCTTCGGTGGT and qRPS9rev TGCTTATTCTTCATCCTCTTCATC) reference gene (Genbank accession AJ749993.1). All amplifications were performed in triplicate and repeated at least on two independent biological repeats.

### Publicly available datasets and *de novo* transcriptome assembly

Sequencing files of *C. roseus* samples (project accessions: SRP035766, SRP005953, SRP017832, SRP041695 and SRP008096) were downloaded from the ftp server of the NCBI SRA database. Exhaustive description of all files is provided in Additional file 3: Table S1. Files were converted to fastq files with the NCBI SRA toolkit (v2.3.4-2) and checked with FastQC (v0.11.2). Overall quality was good (Additional file 4: Table S2) but reads from left and right sequencing of paired-end samples as well as single end samples were treated to remove aberrant fragments and adaptors with Trimmomatic v.0.32 [67]. Parameters were the following: Illuminaclip = 2:30:10, Leading = 3, Trailing = 3, Sliding Window = 4:15 and Minimum Length = 36. Adapter sequences were trimmed according to the library design used (GIIx or HiSeq2000). Correct reads were subsequently subjected to *in silico* normalization after being converted into the fasta format with Fastools. Trinity’s *in silico* normalization (v2.0.4) relies on the processing of a *k*-mer library (with Jellyfish v2.1.4, *k* = 25) obtained from reads and was used to discard reads having aberrant k-mer abundance and those which coverage (abundance in a given transcript) exceeded 50 (max_cov = 50). This step aims at reducing overrepresented reads that may impede the reconstruction process. Detailed description of the number of processed reads (trimmed and normalized) is available in Additional file 4: Table S2. Paired-end normalized samples were then assembled with Trinity (v2.0.4) [46, 62]. For testing purposes, Trinity was also performed on all the reads combined from ever paired-end normalized sample, with respecting read orientation. Before processing this large number of reads, a second normalization step was performed with the same parameters as described above. Parameters for Inchworm, Chrysalis and Butterfly were defaults. All the steps were conducted on the CCSC computer grid facility (Orléans, France) using the SLURM scheduler running under on a Linux ×86_64 architecture.

### Clustering of similar sequences

Different homology thresholds (−c parameter, word length–n 9) were tested for CD-HIT-EST [54] (multithreaded revised version 784a6f1b5e11 which supports longer fragments) to evaluate the ability to combine similar sequences while preserving isoforms from being assembled in a same contig. This program returns a ‘.clstr’ files containing the contig composition of each cluster and a multi-fasta file containing the representative sequences of each cluster. A representative sequence is the contig which matched best the other contigs found in the same cluster.

### Sequence alignment and annotation

BLAST analyses against given databases were performed with the stand alone application v2.29 [68]. Annotation of transcripts was performed with Blastx against the UniprotKB/Swiss-prot database and analyze of hit coverage was done with Trinity perl script analyze_blastPlus_topHit_coverage.pl. This analysis focuses on the number of proteins which are matched at least once by sequences in a given transcriptome [62]. Quick evaluation of transcriptome assemblies was done by setting a local BLAST server with SequenceServer (Pryiam et al., unpublished) and analyzing candidate sequences from the MIA pathway with Blastn.

### Estimation of transcript abundance

Estimation of transcript abundance was performed with RSEM v1.2.15 after aligning reads to target transcriptome with Bowtie2 [63] (v2.2.5) with default parameters for both programs and the–no-polyA option. FPKM for the CD97 and CDF97 datasets were computed after preparing respective contig-to-cluster maps (using ‘.clstr’ file generated with CD-HIT-EST) for the rsem-prepare-reference procedure in order to get expression values reflecting abundance in all samples. This is expected to allow alignment of cultivar-specific reads to its cognate contig, while using its expression level for the whole cluster. Expression tables for CD97 and CDF97 were prepared by merging ‘.genes.results’ files. To compare expression levels between qPCR and FPKM in other assemblies, expression levels were re-calculated using the above described procedure but without contig-to-cluster or transcript-to-gene information.

### Data processing

The R software (3.1.0, [69]) was used with the GUI interface RStudio v0.98.1091 or in the command line interface for high multicore parallelization. All operations outside dedicated programs were performed with R. The Bioconductor package ‘SRA.db’ [70] (v1.22.0) was used to retrieve sample information from the SRA. We used the ‘seqinr’ [71] package (v3.1-3) to remove poorly represented clusters from CD97 after identification of appropriate thresholds for cluster length, abundance and contig number. Graphs for the transcriptomic analysis were generated with the ‘ggplot2’ package [72] (v1.0.1). Correlations between qPCR and FPKM data were calculated as the adjusted r^2^ of a linear model built with the “lm” function. The correlation was established for each dataset using their own expression data (see above).

## Availability of supporting data

The dataset (CDF97) supporting the results of this article is available at the LabArchives, LLC, repository, with DOI number 10.6070/H4DR2SG9 and open access at http://dx.doi.org/10.6070/H4DR2SG9. It is also freely available on our website http://bbv-ea2106.sciences.univ-tours.fr/. The non-filtered dataset (CD97) as well as all new assemblies described in this study are available upon request.

## Additional files

Additional file 1: Figure S1. Alignment of the amino-acid sequence of SLS1 and SLS2. Sequence identity and similarity are highlighted by black and grey shading, respectively. Red bars denote the position of a predicted transmembrane helix as described in [36]. Additional file 2: Figure S2. Identification of secoxyloganin in enzymatic assays (left) by comparison with a pure authentic standard using UV spectrum (A) and MS spectra in negative (B) and positive (C) modes. Additional file 3: Table S1. Details for each RNA-seq sample used in this study. Additional file 4: Table S2. Quality control (FastQC) of paired-end samples, number of reads before and after trimming/normalization procedures and characteristics of individual assemblies. Additional file 5: Figure S3. Quality of reconstruction of MIA genes in the assembly constructed with reads from all 19 paired-end samples. As a large number of sequences were obtained for this assembly (353,245), redundant sequences were clustered with CD-HIT-EST using different % identity thresholds; A = ccOrcae, B = mpgrCra, C = NIPGR, D = PMS454, E = PMSIllu, no = no clustering.

## Abbreviations

MIA: Monoterpenoid indole alkaloids
TDC: Tryptophan decarboxylase
MEP: Methyl-erythritol phosphate
GAP: Glyceraldehyde 3-phosphate
IPP: Isopentenyl diphosphate
DMAPP: Dimethylallyl diphosphate
GPP: Geranyl diphosphate
GES: Geraniol synthase
G10H: Geraniol 10-hydroxylase
10HGO: 10-Hydroxygeraniol oxidoreductase
IS: Iridoid synthase
IO: Iridoid oxidase
UGT: UDP-glucose glycosyltransferase
7DLGT: 7-Deoxyloganetic acid glucosyltransferase
7DLH: 7-Deoxyloganic acid 7-hydroxylase
LAMT: S-adenosyl-L-methionine: loganic acid methyl transferase
SLS: Secologanin synthase
STR: Strictosidine synthase
SGD: Strictosidine β-D-glucosidase
T16H: Tabersonine 16-hydroxylase
16OMT: 16-Hydroxytabersonine O-methyltransferase
NMT: 16-Methoxy-2,3-dihydrotabersonine N-methyltransferase
D4H: Desacetoxyvindoline-4-hydroxylase
DAT: Deacetylvindoline-4-*O*-acetyltransferase
IPAP: Internal phloem associated parenchyma
MPGR: Medicinal plant genomics resource
CC: Cathacyc
PMS: Phytometasyn
UPLC-MS: Ultra-performance liquid chromatography-mass spectrometry
YFP: Yellow fluorescent protein
FPKM: Fragments per kilobase of exon per million fragments mapped
UTR: Untranslated region
