# Supplementary material for: Characterization of a second secologanin synthase isoform producing both secologanin and secoxyloganin allows enhanced de novo assembly of a Catharanthus roseus transcriptome
Source: BMC Genomics. 2015 Aug 19;16(1):619. doi: 10.1186/s12864-015-1678-y (PMC4541752; doi:10.1186/s12864-015-1678-y)
Supplement: Additional file 1: Figure S1. — Alignment of the amino-acid sequence of SLS1 and SLS2. Sequence identity and similarity are highlighted by black and grey shading, respectively. Red bars denote the position of a predicted transmembrane helix as described in [36]. [file 12864_2015_1678_MOESM1_ESM.docx]

SLS1 1 MEMDMDTIRKAIAATIFALVMAWAWRVLDWAWFTPKRIEKRLRQQGFRGNPYRFLVGDVK
SLS2 1 MEMDMDIIRKAIAATIFALVMAWAWRVLDWAWFTPKRIEKRLRQQGFRGNPYRFLVGDVK

SLS1 61 ESGKMHQEALSKPMEFNNDIVPRLMPHINHTINTYGRNSFTWMGRIPRIHVMEPELIKEV
SLS2 61 ESGKMHQEALSNPMEFDNDIVPRLMPHINHTIKTYGRNSFTWMGRIPRIHVMEPELIKEV

SLS1 121 LTHSSKYQKNFDVHNPLVKFLLTGVGSFEGAKWSKHRRIISPAFTLEKLKSMLPAFAICY
SLS2 121 LTHSSKYQKNFDVHNPLVKFLLTGVGSFEGAKWSKHRRIISPAFTLEKLKSMLPAFAICY

SLS1 181 HDMLTKWEKIAEKQGSHEVDIFPTFDVLTSDVISKVAFGSTYEEGGKIFRLLKELMDLTI
SLS2 181 HDMLTKWEKLAEKEGSHEVDIFPTFDVLTSDVISKVAFGSTYDEGGKIFRLLKELMDLTI

SLS1 241 DCMRDVYIPGWSYLPTKRNKRMKEINKEITDMLRFIINKRMKALKAGEPGEDDLLGVLLE
SLS2 241 DCMRDVYIPGWSYLPTKRNKRMKEINKEITDMLRFIINKRMKALKAGEPGEDDLLGVLLE

SLS1 301 SNIQEIQKQGNKKDGGMSINDVIEECKLFYFAGQETTGVLLTWTTILLSKHPEWQERARE
SLS2 301 SNIQEIQKQGNRKDGGMTINDVIEECKLFYFAGQETTGVLLTWTTILLSKHPEWQERARE

SLS1 361 EVLQAFGKNKPEFERLNHLKYVSMILYEVLRLYPPVIDLTKIVHKDTKLGSYTIPAGTQV
SLS2 361 EVLQAFGKNKPEFERLNHLKYVSMILYEVLRLYPPVIDLTKIIHEDTKLGPYTIPAGTQV

SLS1 421 MLPTVMLHREKSIWGEDAMEFNPMRFVDGVANATKNNVTYLPFSWGPRVCLGQNFALLQA
SLS2 421 MLPTVMLHREKSIWGEDAMEFNPMRFADGVANATKNNVTYLPFSWGPRVCLGQNFALLQA

SLS1 481 KLGLAMILQRFKFDVAPSYVHAPFTILTVQPQFGSHVIYKKLES---
SLS2 481 KLGLAMILQRFKFDVAPSYVHAPFTILTVQPQFGSHVIYKKLERQNF


**Supplemental Figure 1. Alignment of the amino-acid sequence of SLS1 and SLS2.** Sequence identity and similarity are highlighted by black and grey shading, respectively. Red bars denote the position of a predicted transmembrane helix as described in [36].
