# Supplementary material for: Characterization of a second secologanin synthase isoform producing both secologanin and secoxyloganin allows enhanced de novo assembly of a Catharanthus roseus transcriptome
Source: BMC Genomics. 2015 Aug 19;16(1):619. doi: 10.1186/s12864-015-1678-y (PMC4541752; doi:10.1186/s12864-015-1678-y)
Supplement: Additional file 2: Figure S2. — Identification of secoxyloganin in enzymatic assays (left) by comparison with a pure authentic standard using UV spectrum (A) and MS spectra in negative (B) and positive (C) modes. [file 12864_2015_1678_MOESM2_ESM.pptx]

## Slide 1
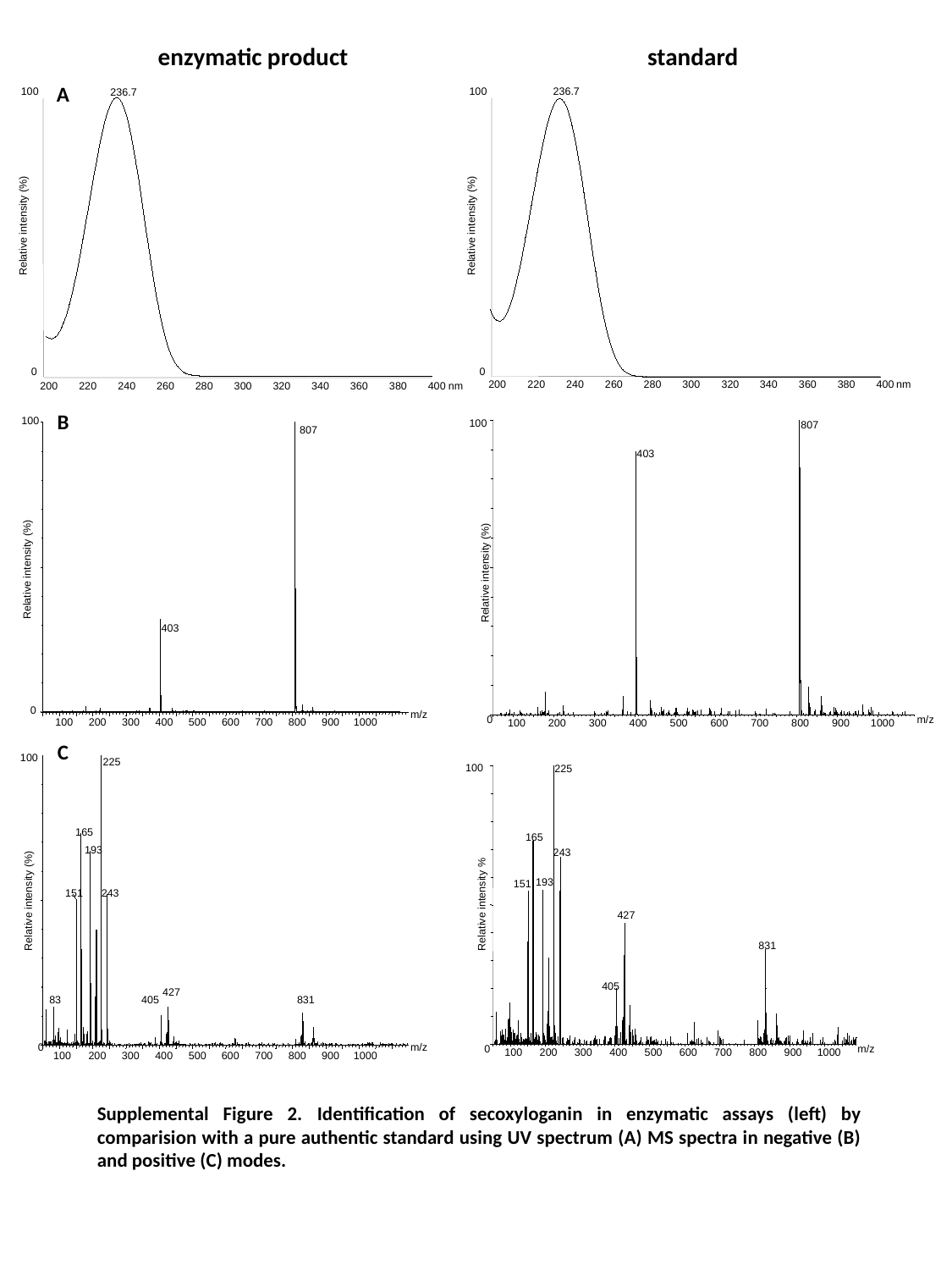

enzymatic product
standard
A
236.7
100
100
236.7
Relative intensity (%)
0
200
220
240
260
280
300
320
340
360
380
400
Relative intensity (%)
0
200
220
240
260
280
300
320
340
360
380
400
nm
nm
100
Relative intensity (%)
0
100
200
300
400
500
600
700
800
900
1000
m/z
B
100
Relative intensity (%)
0
m/z
100
200
300
400
500
600
700
800
900
1000
807
403
807
403
C
100
225
165
193
243
151
Relative intensity (%)
427
83
405
831
0
m/z
100
200
300
400
500
600
700
800
900
1000
100
Relative intensity %
0
m/z
100
200
300
400
500
600
700
800
900
1000
225
165
243
193
151
427
831
405
Supplemental Figure 2. Identification of secoxyloganin in enzymatic assays (left) by comparision with a pure authentic standard using UV spectrum (A) MS spectra in negative (B) and positive (C) modes.
