# Supplementary figures and images for: Characterization of a second secologanin synthase isoform producing both secologanin and secoxyloganin allows enhanced de novo assembly of a Catharanthus roseus transcriptome
Source: BMC Genomics. 2015 Aug 19;16(1):619. doi: 10.1186/s12864-015-1678-y (PMC4541752; doi:10.1186/s12864-015-1678-y)

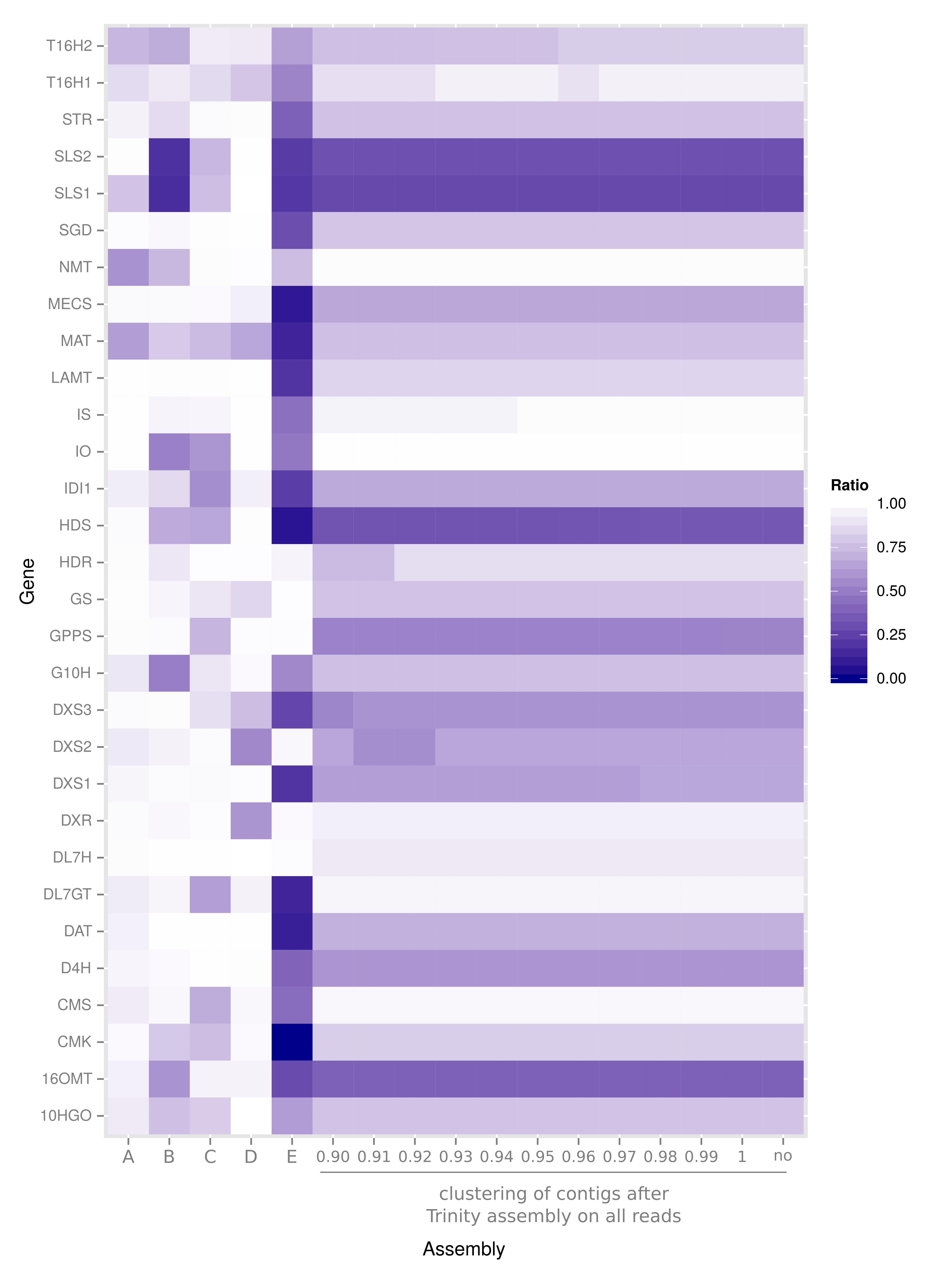

Supplement: Additional file 5: Figure S3. — Quality of reconstruction of MIA genes in the assembly constructed with reads from all 19 paired-end samples. As a large number of sequences were obtained for this assembly (353,245), redundant sequences were clustered with CD-HIT-EST using different % identity thresholds; A = ccOrcae, B = mpgrCra, C = NIPGR, D = PMS454, E = PMSIllu, no = no clustering. [file 12864_2015_1678_MOESM5_ESM.png]
